# Early Change in Area Strain Detected by 3D Speckle Tracking Is Associated With Subsequent Cardiotoxicity in Patients Treated With Low Doses of Anthracyclines

**DOI:** 10.3389/fcvm.2022.842532

**Published:** 2022-03-21

**Authors:** Rafael B. Piveta, Ana Clara T. Rodrigues, Marcelo L. C. Vieira, Cláudio H. Fischer, Tania R. Afonso, Edgar Daminello, Felipe M. Cruz, Tatiana F. G. Galvão, Edgar B. L. Filho, Marcelo Katz, Samira S. Morhy

**Affiliations:** ^1^Department of Echocardiography, Hospital Israelita Albert Einstein, São Paulo, Brazil; ^2^Department of Chemotherapy, Instituto Brasileiro de Controle do Câncer, São Paulo, Brazil

**Keywords:** cardiotoxicity, chemotherapy, echocardiography, 3D strain, 3D speckle tracking

## Abstract

**Objective:**

To evaluate the prognostic impact of the parameters of myocardial deformation using three-dimensional speckle tracking echocardiography (3DSTE) in patients with breast cancer who underwent chemotherapy with low doses of anthracyclines.

**Background:**

Chemotherapy-related cardiotoxicity has an important prognostic impact on cancer survivors. Three-dimensional STE has revealed more consistent data than two-dimensional techniques and may represent a more accurate tool in the evaluation of myocardial function in patients who underwent chemotherapy.

**Methods:**

We evaluated patients with breast cancer who were treated with anthracyclines (associated or not with trastuzumab) in five stages: baseline, after cumulative doses of 120 and 240 mg/m^2^ of doxorubicin, and then, after 6 months and at least 1 year after anthracyclines. Ultrasensitive troponin I (US-TnI) and a standard echocardiography study were performed at each stage. We analyzed left ventricular ejection fraction (LVEF) by Simpson's method, two-dimensional speckle tracking (2DSTE) with longitudinal and radial strain values, and 3DSTE with longitudinal, radial, and circumferential strain as well as twist, torsion, rotation, and three-dimensional global area strain (3DGAS). Cardiotoxicity was defined as a decrease in LVEF by more than 10 percentage points to a value lower than 53%.

**Results:**

We evaluated 51 female patients who were aged 50.6 ± 11 years. After the cumulative dose of 240 mg/m^2^ of doxorubicin, US-TnI was increased (>34 pg/ml) in 21 patients (45%, *p* > 0.001), LVEF remained unchanged (*p* = 0.178), while 2DSTE longitudinal strain was decreased (from −17.8% to −17.1%, *p* < 0.001) and 3DSTE detected changes in longitudinal, radial, circumferential, and area strain. After a lower cumulative dose of doxorubicin (120 mg/m^2^), 3DGAS (*p* < 0.001) was the only parameter that was changed. In the follow-up, 7 (13%) patients presented a decrease in LVEF. Three-dimensional GAS early changed to abnormal values was the only variable associated with a subsequent decrease in LVEF (definitive cardiotoxicity).

**Conclusion:**

In patients with breast cancer, 3DSTE detected early changes in area strain after very low doses of doxorubicin. The 3DGAS early changed to abnormal values was associated with a subsequent decrease in LVEF, representing a promising technique to predict chemotherapy-induced cardiomyopathy.

## Background

Advances in oncology therapy have increased cancer patient survival rates (1). However, these patients are exposed to the damaging effects of cancer-therapeutic related cardiac dysfunction (CTRCD), which represents a significant cause of morbidity and mortality (2, 3). This complication may result in cancer treatment discontinuation and compromise cancer control or its cure (4). Additionally, chemotherapy-related heart failure (HF) often has a worse prognosis than many cancers, with mortality as high as 60% within 2 years (2). Early detection of cardiotoxicity along with cardioprotection therapy is fundamental to improve the prognosis of these patients (5, 6). However, the usual parameters used for this diagnosis, especially left ventricular ejection fraction (LVEF), have low sensitivity, changing only in the later phases, when the majority of patients do not respond to treatment (5, 7). Thus, there is increased interest in identifying early markers of cardiotoxicity that could predict the subsequent decrease in LVEF and progression to HF.

In this setting, the myocardial deformation assessed by two-dimensional speckle-tracking echocardiography (2DSTE), especially two-dimensional global longitudinal strain (2DGLS), has been demonstrated to play a significant role in the diagnosis of subclinical cardiotoxicity (8–11). Recent expert consensus strongly supports a 2DGLS-based follow-up of adults during and after cancer therapy, and a reduction of this parameter by >15% is likely to be of clinical significance, since it might predict a decrease in LVEF (12, 13). However, the 2DSTE technique presents limitations, which may impair analysis of cardiac mechanics (14).

Three-dimensional echocardiography provides a greater proximity to the cardiac anatomy and a high accuracy and reproducibility as compared to cardiac magnetic resonance (CMR) (15). An important advance was the development of the three-dimensional speckle tracking echocardiography (3DSTE), which has overcome many limitations related to 2DSTE (16, 17). The 3DSTE does not rely on geometric assumptions, and the speckles are tracked by means of the homogeneous spatial distribution of each component of the myocardial displacement vector (16, 17). Previous data suggest that when compared to the 2DSTE, 3DSTE has a higher correspondence with measures of cardiac mechanics, greater accuracy, and efficiency (16, 17). Although reproducibility and applicability of 3DSTE have already been demonstrated in different clinical settings (18–21), this technique has been poorly explored in cancer patients who underwent chemotherapy.

This prospective study evaluated myocardial deformation using 2DSTE and 3DSTE in patients with breast cancer who underwent chemotherapy with low doses of anthracyclines, to identify early markers of cardiotoxicity and its association with a subsequent decrease in LVEF or development of HF.

## Methods

### Study Population

In this prospective cohort, patients at least 18 years old, newly diagnosed with breast cancer, and scheduled to receive chemotherapy with anthracyclines were eligible for the study.

Exclusion criteria included previous treatment with chemotherapy or radiotherapy, inadequate echocardiographic image, and the presence of left ventricular systolic dysfunction with an LVEF <55% before chemotherapy.

The Ethics Committee approved the study, and written informed consent was obtained from all patients.

Cancer treatment was scheduled in four chemotherapy cycles with a 21-day interval between them. In each cycle, 60 mg/m^2^ of non-liposomal doxorubicin was administered, totaling 240 mg/m^2^, together with 600 mg/m^2^ of cyclophosphamide, totaling 2, 400 mg/m^2^. Human epidermal growth factor receptor 2 (HER-2) positive patients received trastuzumab treatment. In selected cases, patients received cisplatin, paclitaxel, and/or radiotherapy.

**Figure 1:**
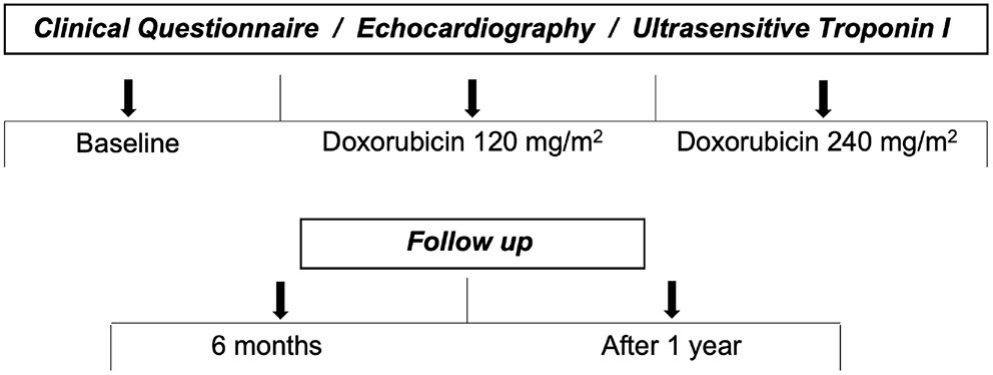
Stages of evaluation.

Patients underwent a comprehensive echocardiographic study and laboratory collection of serum ultrasensitive troponin I (US-TnI) during the following 5 stages: baseline, after cumulative doses of 120 and 240 mg/m^2^ of doxorubicin, and then, after 6 months and at least 1 year after anthracyclines (Figure 1). The echocardiographic and laboratory analyses were performed between 3 and 7 days after exposure to the anthracyclines.

Based on current guidelines, cardiotoxicity was defined as a decrease in LVEF by more than 10 percentage points to a value lower than 53% (12, 13).

### Standard Echocardiography

Echocardiography was performed using Artida 4D equipment (Toshiba Medical Systems Corporation, Otawara-shi, Japan). Image acquisition and analysis were performed by one experienced echocardiographer who was blinded to prior echocardiographic results. At least three consecutive cardiac cycles were obtained, and the exams were digitally stored for subsequent analysis. LVEF assessed was measured using the Simpson biplane method. From the apical 4-chamber view, E wave velocity, A wave velocity, and E/A ratios were measured using conventional Doppler; peak systolic (s′), early diastolic (e′) mitral annular septal, and lateral velocities were measured using tissue Doppler imaging; and the E/e′ ratio was also calculated. Analysis of LV diastolic function was performed according to the recommendations of the American and European Societies of Echocardiography (22). Right ventricular systolic function was also evaluated.

### Speckle Tracking Echocardiography

Peak systolic strain was measured with 2DSTE at a frame rate of 50–90 frames/s or ≥40% of the heart rate. The adequacy of tracking was verified manually, and the region of interest was readjusted to achieve optimal tracking if necessary. Two-dimensional GLS was calculated by averaging the peak systolic strain values in the myocardial segments of the apical 2-, 3-, and 4-chamber views. Additionally, two-dimensional radial strain (2DRS) was calculated by averaging peak systolic strain values in all 6 segments of the parasternal short-axis view at the midpapillary level.

The 3DSTE technique was performed from the apical 4-chamber view (full volume acquisition) using a fully sampled matrix array transducer (PST-25SX). The whole LV was positioned in the pyramidal volume while the depth and width of the sector were reduced to improve spatial and temporal resolution (20–30 volumes/s). After the acquisition, views in five planes in the standard axis were exposed, two longitudinally oriented orthogonally and three transversally oriented. The strain analysis was undertaken based on the marking of three points on the LV myocardium. The endocardial border was detected automatically by the 3D Wall Motion Tracking software (Toshiba Medical Systems Corporation, Otawara-shi, Japan). The tracings were modified manually only in areas where the endocardial borders were not adequately marked. The strain was automatically calculated by the software during the whole cardiac cycle, providing continuous values for 17 myocardial segments simultaneously. The mechanical analysis derived from the 3DSTE was allowed for the following parameters to be calculated: three-dimensional global longitudinal strain (3DGLS), three-dimensional global radial strain (3DGRS), three-dimensional global circumferential strain (3DGCS), three-dimensional global area strain (3DGAS), rotation, torsion, and twist.

### Reproducibility

Interobserver and intraobserver variabilities were evaluated in randomized patients using an intraclass correlation coefficient with 95% CIs.

#### Ultrasensitive Troponin I

The analysis of US-TnI was undertaken with the immunoassay method (chemoluminescence) and expressed in pg/ml, using the Vitros® 5600 (Ortho Clinical Diagnostics, Johnson & Johnson) equipment. Values above 34 pg/ml were considered to be increased.

#### Statistical Analysis

Continuous data were presented as mean ± SD or median with interquartile range. Categorical data were presented as percentages.

The echocardiographic parameters and the US-TnI values were described according to evaluation stages and compared with generalized estimating equations with normal distribution and identity link function, supposing a matrix of autoregressive correlations of the first-order between the moments. For US-TnI, the logarithmic correction function was used due to the asymmetric distribution of the values. The analyses were followed by multiple Bonferroni comparisons when appropriate.

The follow-up changes in LVEF were created after the use of chemotherapy and verified who suffered and who did not change and compared the deltas of the cardiac parameters of patients according to change in LVEF using Student's t-tests and verified the association of qualitative characteristics with LVEF alteration using Fisher's exact tests or likelihood ratio tests.

The statistical analysis was performed with IBM-SPSS *for Windows* version 20.0. Values of *p* < 0.05 were considered significant.

## Results

### Study Population

Sixty-eight patients were eligible for the study, with seventeen exclusions (six due to inadequate echocardiographic image, five because they did not wish to follow-up, and two non-cardiac-related death). Accordingly, 51 patients underwent echocardiographic and laboratory analysis at baseline, after cumulative doses of 120 and 240 mg/m^2^ of doxorubicin, and then, after 6 months and at least 1 year after anthracyclines. After a cumulative dosage of 120 mg/m^2^ of doxorubicin, two patients did not present for evaluation due to fatigue and in the stage just after 6 months of treatment, 4 patients did not present for evaluation because they did not wish to follow-up. The mean follow-up time was 1.6 ± 0.6 years.

All patients were women with a mean age of 50 ± 11 years. The clinical characteristics are summarized in Table 1. All patients were treated with a cumulative total dose of 240 mg/m^2^ of doxorubicin and 2, 400 mg/m^2^ of cyclophosphamide, with an intravenous infusion time of 2 h per cycle. Seven patients were treated with trastuzumab and 45 of them with paclitaxel (Table 2).

**Table 1:** Baseline clinical characteristics of the patients.

**Variable**	***n =* 51**
Age (years)	50.6 ± 11.3
Body mass index (kg/m^2^) Heart rate (beats/min)	26.8 ± 4.9 75.8 ± 9
**Cardiovascular Risk Factors** ***n*** **(%)**	
Diabetes	6 (11.8)
Hypertension	13 (25.5)
Hyperlipidemia	2 (3.9)
Smoking	6 (11.8)
Hypothyroidism	2 (3.9)
Overweight/Obesity	12 (23.5)
Previous Heart Disease	0 (0)
**Cardiovascular treatment** ***n*** **(%)**	
ACE inhibitor/ARB	7 (13.7)
Beta-Blocker	6 (11.8)
Diuretics	3 (5.9)
Statins	1 (2)
Diabetes treatment	6 (11.8)

*ACE, angiotensin-converting enzyme; ARB, angiotensin II receptor blocker*.

*Data are expressed as the mean ± SD or as number (percentage)*.

**Table 2:** Cancer treatment and metastatic stage.

**Variable**	**(*n =* 51)**
Doxorubicin	51 (100%)
Cyclophosphamide	51 (100%)
Trastuzumab	7 (13.7)
Paclitaxel	45 (88.2)
Cisplatin	1 (2)
Radiotherapy	45 (88.2)
Surgery	28 (54)
Metastatic Stage	2 (3.9)

*Data are expressed as number (percentage)*.

None of the patients presented HF symptoms. After beginning chemotherapy, 13 patients (25%) presented non-specific fatigue.

### Echocardiography

All echocardiographic parameters in the 5 evaluation stages are presented in Table 3.

**Table 3:** Echocardiographic parameters in the 5 evaluation stages.

**Variable**	**Baseline**	**120 mg/m^**2**^**	**240 mg/m^**2**^**	**6 months**	**1 year**	***p*-value**
Heart rate (beat/min)	75.8 ± 9.1	76.1 ± 10.3	75.3 ± 10.5	75.1 ± 9.6	71.5 ± 10.6	0.067
LVEF	0.64 ± 0.02	0.63 ± 0.03	0.63 ± 0.03	0.63 ± 0.03	0.60 ± 0.04	0.001
Diastolic dysfunction^†^	17 (33.3%)	22 (44%)	22 (44%)	17 (33.3%)	24 (47%)	0.169
E-wave (cm/s)	81.3 ± 18	82.2 ± 18.5	80.5 ± 19.6	83.5 ± 17.6	86.5 ± 18.6	0.455
A-wave (cm/s)	74.2 ± 19.9	75.8 ± 21.7	77.2 ± 20.3	76.8 ± 21.8	74.2 ± 21.3	0.234
DT (ms)	192 ± 42.2	190.5 ± 39.7	180 ± 45.1	207 ± 59.1	208 ± 46.2	0.060
E/A ratio	1.2 ± 0.4	1.1 ± 0.4	1.1 ± 0.4	1.1 ± 0.3	1.1 ± 0.4	0.120
Septal s′ (cm/s)	9.2 ± 1.4	9.2 ± 1.3	9.1 ± 1.3	8.8 ± 1.7	8.4 ± 1.2	0.058
Septal e′ (cm/s)	10.7 ± 3.0	10.2 ± 3.5	9.6 ± 2.6	9.6 ± 3.7	9.0 ± 2.5	0.021
Lateral s′ (cm/s)	10.8 ± 2.9	10.3 ± 2.0	10 ± 2.1	9.2 ± 1.9	8.9 ± 1.8	0.001
Lateral e′ (cm/s)	13.5 ± 4.2	12.8 ± 4.2	12.7 ± 3.6	13.1 ± 4.1	12.1 ± 4.2	0.247
E/e′ ratio	7.37 ± 1.6	7.1 ± 1.9	7.1 ± 2.2	7.4 ± 1.9	7.5 ± 1.8	0.617
RV s′ (cm/s)	13.7 ± 1.9	13.6 ± 2.4	13.5 ± 2.4	12.7 ± 2.1	12.5 ± 2.0	0.108
TAPSE (mm)	20.6 ± 4.1	19.9 ± 2.3	20.2 ± 2.4	20.3 ± 2.3	20.7 ± 2.9	0.635
**2D Speckle Tracking**						
2DGLS (%)	−17.8 ± 1.5	−17.4 ± 1.4	−17.1 ± 1.4	−17.0 ± 2.2	−16.6 ± 2.1	<0.001
2DRS (%)	39.3 ± 9.8	36.7 ± 8.6	36.3 ± 14	35.1 ± 12.1	36.5 ± 13.4	0.516
**3D Speckle Tracking**						
3DGLS (%)	−15.9 ± 2.2	−15.5 ± 2.2	−14.8 ± 1.9	−14.1 ± 6.3	−14.4 ± 3.2	0.043
3DGRS (%)	31.7 ± 12.8	27.5 ± 10.3	26.3 ± 11.6	26.2 ± 11.1	30 ± 13.1	0.043
3DGCS (%)	−33.8 ± 4.7	−32.3 ± 5^†^	−31.2 ± 4.2	−30.4 ± 6.3	−30.1 ± 6.5	0.023
*Twist* (°)	6.1 ± 2.8	5.3 ± 3	4.8 ± 2.4	4.5 ± 2.5	5 ± 4.5	0.278
Torsion (°/cm)	2.6 ± 1.4	2.5 ± 1.5	2.4 ± 1.3	1.9 ± 1.1	1.9 ± 1.5	0.068
Rotation (°)	5.2 ± 2.9	5.1 ± 3.4	4.5 ± 2.2	4.4 ± 2.1	3.8 ± 2	0.093
3DGAS (%)	−45.4 ± 4.1	−43.2 ± 4.4	−39.8 ± 3.3	−39 ± 3.1	−39 ± 2.9	<0.001

*GAS, global area strain; GLS, global longitudinal strain; GCS, global circumferential strain; GRS, global radial LVEF, left ventricular ejection fraction; DT, E-wave deceleration time; RV, right ventricle; TAPSE, tricuspid annular plane systolic excursion; data are expressed as the mean ± SD or as number (percentage). Comparisons made by generalized estimation equations with normal distribution and identity link function*.

† *Generalized equations with binomial distribution and logit bond function. p < 0.05 was considered significant*.

**Figure 2:**
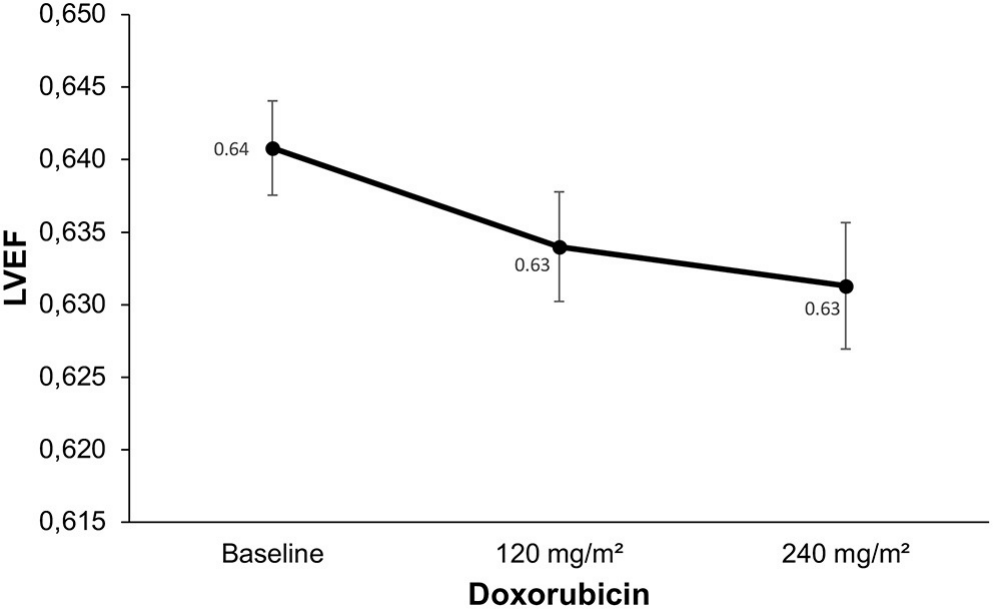
Changes in left ventricle ejection fraction (LVEF) during the treatment. Compared to baseline, LVEF did not change during the chemotherapy with doxorubicin (*p* = 0.178).

The standard echocardiographic parameters in the early stages of evaluation (baseline and after 120 and 240 mg/m^2^) during the treatment are summarized in Table 4. Mean LVEF, as well as conventional Doppler parameters, were unchanged during treatment of doxorubicin (Figure 2). Among tissue Doppler indices, only the e′ septal velocity was decreased after treatment with 240 mg/m^2^ of doxorubicin (from 10.7 ± 3.1 cm/s at baseline to 9.6 ± 2.6 cm/s; *p* = 0.019), with no change found for a lower cumulative dosage of doxorubicin (*p* > 0.99). LVEF was changed after the fifth assessment stage (1 year; *p* = 0.001), however, the mean value remained normal. The s′ lateral velocity was decreased after 1 year of chemotherapy (from 10.7 ± 2.9 cm/s at baseline to 8.9 ± 21.8 cm/s after 1 year; *p* = 0.001), however, there was no early change in this parameter (*p* = 0.220; Table 4). No changes were observed for LV diastolic function, valvular function, or right ventricular performance during the stages of evaluation.

**Table 4:** Standard echocardiographic parameters during the treatment.

**Variable**	**Baseline**	**120 mg/m^**2**^**	**240 mg/m^**2**^**	*p*-value^*^
LVEF	0.64 ± 0.02	0.63 ± 0.03	0.63 ± 0.03	0.178
Diastolic Dysfunction^†^	17 (33.3%)	22 (44%)	22 (44%)	0.122
E-wave (cm/s)	81.3 ± 18	82.2 ± 18.5	80.5 ± 19.6	0.466
A-wave (cm/s)	74.2 ± 19.9	75.8 ± 21.7	77.2 ± 20.3	0.262
DT (ms)	192.8 ± 42.2	190.5 ± 39.7	180 ± 45.1	0.269
E/A ratio	1.2 ± 0.4	1.1 ± 0.4	1.1 ± 0.4	0.054
Septal s′ (cm/s)	9.2 ± 1.4	9.2 ± 1.3	9.1 ± 1.3	0.663
Septal e′ (cm/s)	10.7 ± 3.0	10.2 ± 3.5	9.6 ± 2.6	0.019
Lateral s′ (cm/s)	10.8 ± 2.9	10.3 ± 2.0	10 ± 2.1	0.220
Lateral e′ (cm/s)	13.5 ± 4.2	12.8 ± 4.2	12.7 ± 3.6	0.156
E/e′ ratio	7.37 ± 1.6	7.1 ± 1.9	7.1 ± 2.2	0.678
RV s′ (cm/s)	13.7 ± 1.9	13.6 ± 2.4	13.5 ± 2.4	0.937
TAPSE (mm)	20.6 ± 4.1	19.9 ± 2.3	20.2 ± 2.4	0.515
Heart Rate (beat/min)	75.8 ± 9.1	76.1 ± 10.3	75.3 ± 10.5	0.961

*LVEF, left ventricular ejection fraction; DT, E-wave deceleration time; RV, right ventricle; TAPSE, tricuspid annular plane systolic excursion; data are expressed as the mean ± SD or as number (percentage)*.

* *After 240 mg/m^2^ dose of doxorubicin compared to baseline. Comparisons made by generalized estimation equations with normal distribution and identity link function*.

† *Generalized equations with binomial distribution and logit bond function. p < 0.05 was considered significant*.

### Speckle Tracking Echocardiography

**Figure 3:**
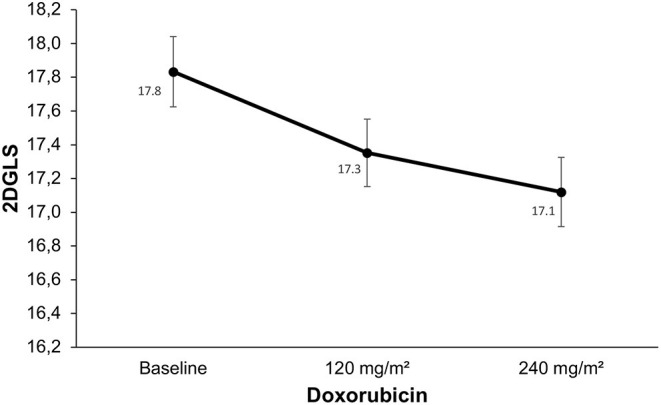
Changes in global longitudinal strain derived from the two-dimensional speckle tracking echocardiography. Compared to baseline, two-dimensional global longitudinal strain (2DGLS) did not change earlier, after 120 mg/m^2^ of doxorubicin (*p* < 0.103). There was a decrease in 2DGLS after 240 mg/m^2^ of doxorubicin compared to baseline (*p* < 0.001). However, the relative change of 2DGLS was mild, with no patient presenting criteria for the diagnosis of subclinical cardiotoxicity.

The 2DGLS presented changes only at the end of the protocol, after the 240 mg/m^2^ dosage (from −17.8% ± 1.5 at baseline to −17.1% ± 1.4; *p* = 0.001; Figure 3 and Table 5), with no change after a lower cumulative dosage of doxorubicin (*p* = 0.103). Two-dimensional RS did not present changes during the evaluation stages (*p* = 0.47).

**Table 5:** Results of myocardial deformation parameters derived from 2DSTE and 3DSTE during the treatment.

**Variable**	**Baseline**	**120 mg/m^**2**^**	**240 mg/m^**2**^**	*p*-value^*^
**2D Speckle Tracking**				
2DGLS (%)	−17.8 ± 1.5	−17.4 ± 1.4	−17.1 ± 1.4	<0.001
2DRS (%)	39.3 ± 9.8	36.7 ± 8.6	36.3 ± 14	0.402
**3D Speckle Tracking**				
3DGLS (%)	−15.9 ± 2.2	−15.5 ± 2.2	−14.8 ± 1.9	<0.002
3DGRS (%)	31.7 ± 12.8	27.5 ± 10.3	26.3 ± 11.6	0.025
3DGCS (%)	−33.8 ± 4.7	−32.3 ± 5^†^	−31.2 ± 4.2	<0.012
Twist (°)	6.1 ± 2.8	5.3 ± 3	4.8 ± 2.4	0.059
Torsion (°/cm)	2.6 ± 1.4	2.5 ± 1.5	2.4 ± 1.3	0.659
Rotation (°)	5.2 ± 2.9	5.1 ± 3.4	4.5 ± 2.2	0.423
3DGAS (%)	−45.4 ± 4.1	−43.2 ± 4.4^†^	−39.8 ± 3.3	<0.001

*GAS, global area strain; GLS, global longitudinal strain; GCS, global circumferential strain; GRS, global radial strain; LVEF, left ventricular ejection fraction; Data expressed as the mean ± SD*.

* *After 240 mg/m^2^ dose of doxorubicin compared to baseline. Comparisons were performed by generalized estimation equations with normal distribution and identity link function*.

† *Multiple comparisons analysis by the Bonferroni method revealing change in 3DGAS after 120 mg/m^2^ dose of doxorubicin compared to baseline (p < 0.001). p < 0.05 was considered significant*.

**Figure 4:**
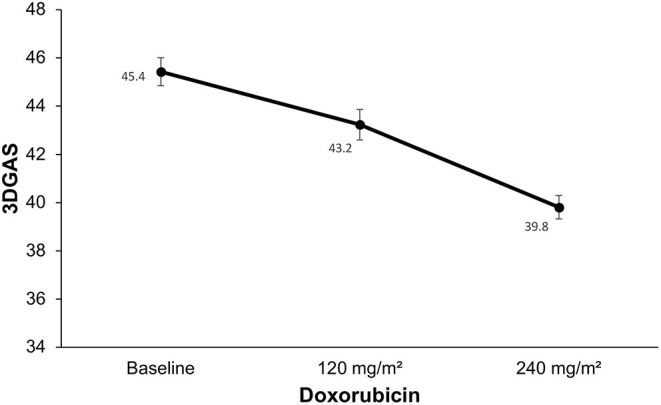
Changes in global area strain derived from the three-dimensional speckle tracking echocardiography. Compared to baseline there was a decrease in three-dimensional global area strain (3DGAS) after a lower cumulative dose of doxorubicin (120 mg/m^2^; *p* < 0.001). There was a decrease in 3DGAS after 240 mg/m^2^ dose of doxorubicin compared to baseline (*p* < 0.001).

After the cumulative dose of 240 mg/m^2^ of doxorubicin, the 3DSTE detected changes in most myocardial deformation parameters: 3DGLS, 3DGRS, 3DGCS, and 3DGAS. There were no changes in rotation, torsion, or twist in any of the evaluation stages (Table 5). Alternatively, after a lower cumulative dose of doxorubicin (120 mg/m^2^), the only parameter that changed was 3DGAS (from −45.4% ± 4.1 at baseline to −43.2% ± 4.1, *p* < 0, 001; Figure 4).

The 3DGAS early changed to below normal values until the period during treatment with doxorubicin was the only parameter that was associated with the subsequent decrease in LVEF during follow-up (*p* = 0.009; Table 6).

**Table 6:** Follow-up. Early changes in variables and clinical features during chemotherapy and its association with a subsequent decrease in LVEF (definite cardiotoxicity).

**Variable**	**Cardiotoxicity**		***p***
	**No (*n =* 44)**	**Yes (*n =* 7)**	
Diabetes			0.578
No	38	7	
Yes	6	0	
Hypertension			>0.999
No	33	5	
Yes	11	2	
ACE inhibitor/ARB			>0.999
No	38	6	
Yes	6	1	
Beta-Blocker			0.186
No	40	7	
Yes	4	0	
Trastuzumab			0.045
No	40	4	
Yes	4	3	
Radiotherapy			>0.999
No	5	1	
Yes	39	6	
US-TnI >34 pg/mL			0.684
No	23	3	
Yes	17	4	
3DGLS (<16.1%)			>0.999
No	10	1	
Yes	30	5	
3DGRS (<24.4%)			0.381
No	26	3	
Yes	14	3	
3DGCS (<28%)			0.057
No	36	6	
Yes	4	0	
3DGAS (<39, 8)			0.009
No	23	0	
Yes	17	7	

*ACE, angiotensin-converting enzyme; ARB, angiotensin II receptor blocker; LVEF, left ventricular ejection fraction; GAS, global area strain; GLS, global longitudinal strain; GCS, global circumferential strain; GRS, global radial strain; US-TnI, ultrasensitive troponin I; data are expressed as number (percentage). Fisher's exact test. p < 0.05 was considered significant. Changes in variables observed during treatment with anthracycline (up to cumulative dose exposure of 240 mg/m^2^ of doxorubicin)*.

In our study, the 3DSTE normality values were based on the main studies that report the normal range values for 3D strain parameters from healthy volunteers (23–25).

### Ultrasensitive Troponin I

The average US-TnI was increased from 12 to 40.7 pg/ml (*p* < 0.001) after the final evaluation stage. Twenty patients (45%) presented abnormal levels of US-TnI (>34 pg/ml) after cumulative dosage of 240 mg/mg^2^ of doxorubicin (*p* = 0.001). After a lower cumulative dose of doxorubicin (120 mg/m^2^), there was no change in US-TnI (*p* = 0.509).

### Follow-Up

In the follow-up, 7 (13%) patients presented a decrease in LVEF with definitive criteria of CTRCD. None of the patients presented HF symptoms.

Table 6 presents the clinical features and the values of the main echocardiographic parameters identified at an early stage (during chemotherapy, up to the stage after 240 mg/m^2^) and their correlation with patients who had a subsequent decrease in LVEF (with definitive cardiotoxicity criteria) in the follow-up.

There was no difference between the clinical characteristics and the presence of cardiovascular risk factors with the development of CTRCD. The use of trastuzumab was slightly associated with a subsequent decrease in LVEF (*p* = 0.045).

Changes in US-TnI during treatment (after cumulative dosage of 240 mg/mg^2^ of doxorubicin) were not associated with a subsequent decrease in LVEF (*p* = 0.684).

The 3DGAS early changed to below normal values was the only parameter that was associated with the subsequent decrease in LVEF during follow-up (*p* = 0.009).

The other mechanical indices derived from 2D and 3D speckle tracking analysis, as well as the conventional echocardiographic parameters were not predictors of CTRCD (Table 6).

### Reproducibility

The intraobserver intraclass coefficients observed were as follows: 2DGLS 0.96 (95% CI, 0.84–0.99), 3DGLS 0.72 (95% CI, 0.21–0.99), 3DGCS 0.97 (95% CI, 0.86–0.99), and 3DGAS 0.99 (95% CI, 0.97–0.99). The corresponding interobserver intraclass coefficients were as follows: 2DGLS 0.92 (95% CI, 0.81–0.98), 3DGLS 0.96 (95% CI, 0.86–0.99), 3DGCS 0.94 (95% CI, 0.82–0.98), and 3DGAS 0.98 (95% CI, 0.92–0.99).

## Discussion

This prospective study shows that 3DSTE may provide added value to the evaluation of left ventricular systolic function in women with breast cancer treated with anthracyclines. The early changes in 3DGAS after low doses of doxorubicin represent an early marker of cardiotoxicity and can identify a subgroup of patients with increased risk for future development of LVEF reduction.

Cardiotoxicity is one of the most important complications of cancer treatment. In this setting, HF has a 2-year mortality rate of up to 60% (2). Anthracyclines-induced cardiotoxicity is mainly mediated through the generation of reactive oxygen species, which may result in cardiomyocyte apoptosis, an irreversible condition (26). Prompt beginning of cardioprotective regimens may interfere in this process and possibly avoid the development of HF, improving prognosis (5, 27). As a result, the search for parameters that allow early identification of patients at risk for future LV dysfunction is of paramount importance.

This study comprised a homogeneous population with comparable cardiovascular risk factors and baseline clinical characteristics. To detect early changes in myocardial mechanics, the study design included patient evaluation after a very low cumulative dosage of doxorubicin (120 mg/m^2^), distinct from previous studies, which, using a similar population, performed the first post-chemotherapy analysis about 3–6 months after exposure to a higher cumulative dosage of doxorubicin (8, 9).

In the present study, the association of trastuzumab was slightly associated with CTRCD, corroborating the fact that the association of drugs in chemotherapy can enhance the occurrence of cardiotoxicity (28).

Myocardial deformation performed by 3DSTE has been shown to be a reliable and reproducible tool for the analysis of LV mechanics in different clinical conditions (18–21). However, this technique has been poorly explored in the scenario of chemotherapy-induced cardiotoxicity. A study evaluated 25 patients with several types of malignancies during chemotherapy and after a 3-month follow-up did not demonstrate changes in any of the 3DSTE indices; however, in this study, only 7 patients were exposed to anthracyclines (29). Another report evaluated 2DSTE and 3DSTE in 55 patients after chemotherapy with anthracyclines and compared to a control group; 3DGAS and 3DGCS were the only parameters that changed. However, in this study, there was no baseline evaluation (30).

In the present study, after the end of treatment, with a cumulative dosage of 240 mg/m^2^ of doxorubicin, changes in several parameters of 3DSTE were observed: 3DGLS, 3DGRS, 3DGCS, and 3DGAS. These changes, found in almost all 3DSTE-derived indices, suggest that the abnormalities caused by reactive oxygen species in cardiomyocytes can be present throughout all directions of myocardial fibers, concordant with experimental models of doxorubicin-induced cardiotoxicity (31). In addition, these changes included radial and circumferential components, which did not prove to be relevant when evaluated by 2DSTE, demonstrating the superiority of the 3D technique over 2D. Several factors, such as the absence of geometric assumptions and no need for multiple plane acquisition, may contribute to that. Additionally, because the speckles move in three dimensions according to the translation movement of the heart, 3DSTE allows a homogeneous spatial distribution of each component of the myocardial displacement vector. Thus, analysis of cardiac mechanics may be technically more accurate with the 3D study (16). It is important to highlight that there are already studies that determine normal values for the LV strain by 3DSTE (23–25).

**Figure 5:**
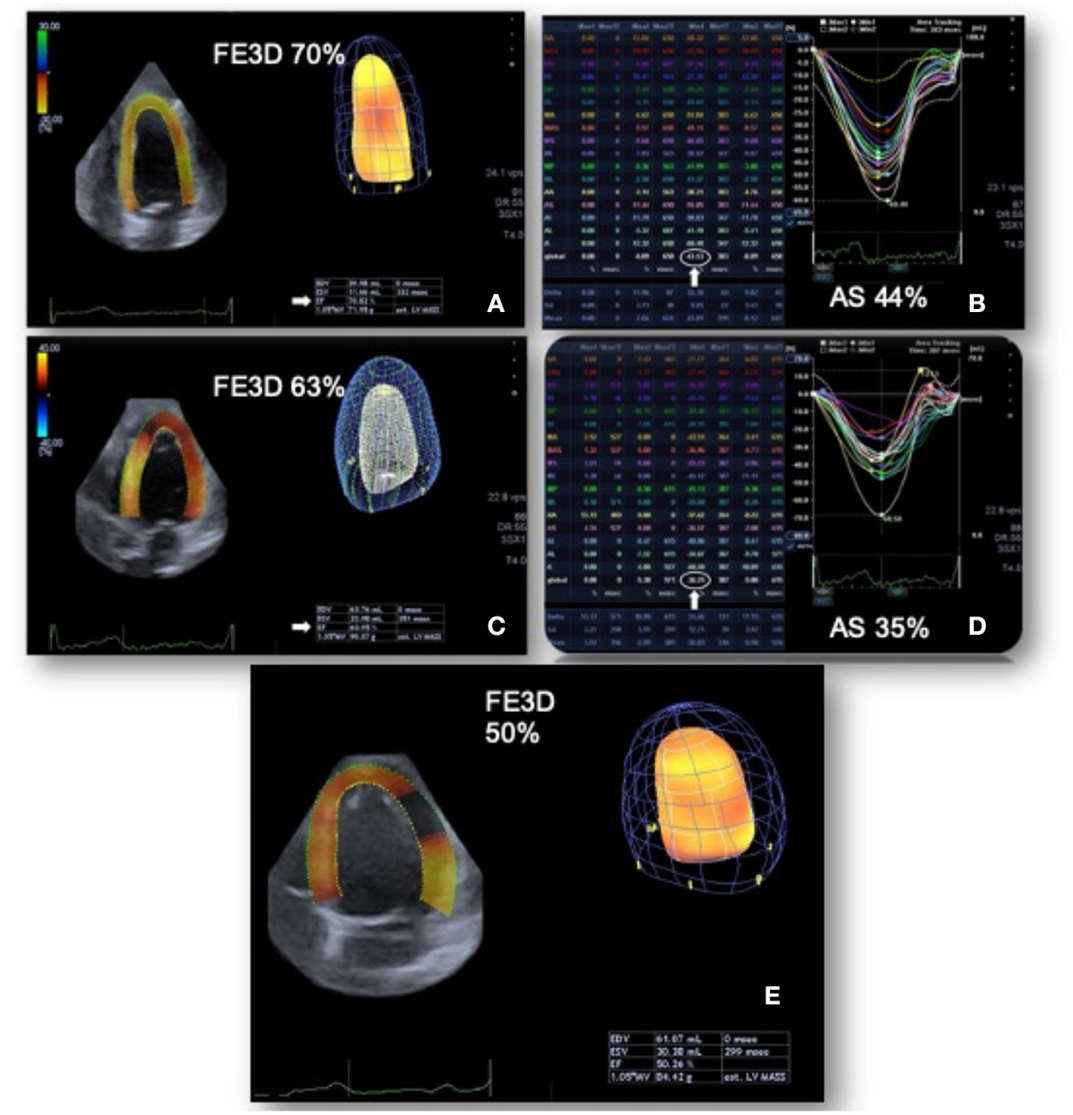
Changes in global area strain derived from the three-dimensional speckle tracking echocardiography. Patient with breast cancer undergoing chemotherapy with doxorubicin. In the baseline evaluation, pre-chemotherapy, left ventricular ejection fraction (LVEF): 0.70 and 3DGAS: −43.5% **(A,B)**. After a low cumulative dose of doxorubicin (120 mg/m^2^), LVEF: 0.63 was observed (with reduction, but still preserved) and a significant decrease in three-dimensional global area strain (3DGAS) to −36.2% **(C,D)**. Six months after the end of chemotherapy with doxorubicin (total cumulative dose of 240 mg/m^2^), the LVEF dropped to 0.50 **(E)**. GAS, global area strain; LVEF, Left ventricle ejection fraction.

**Figure 6:**
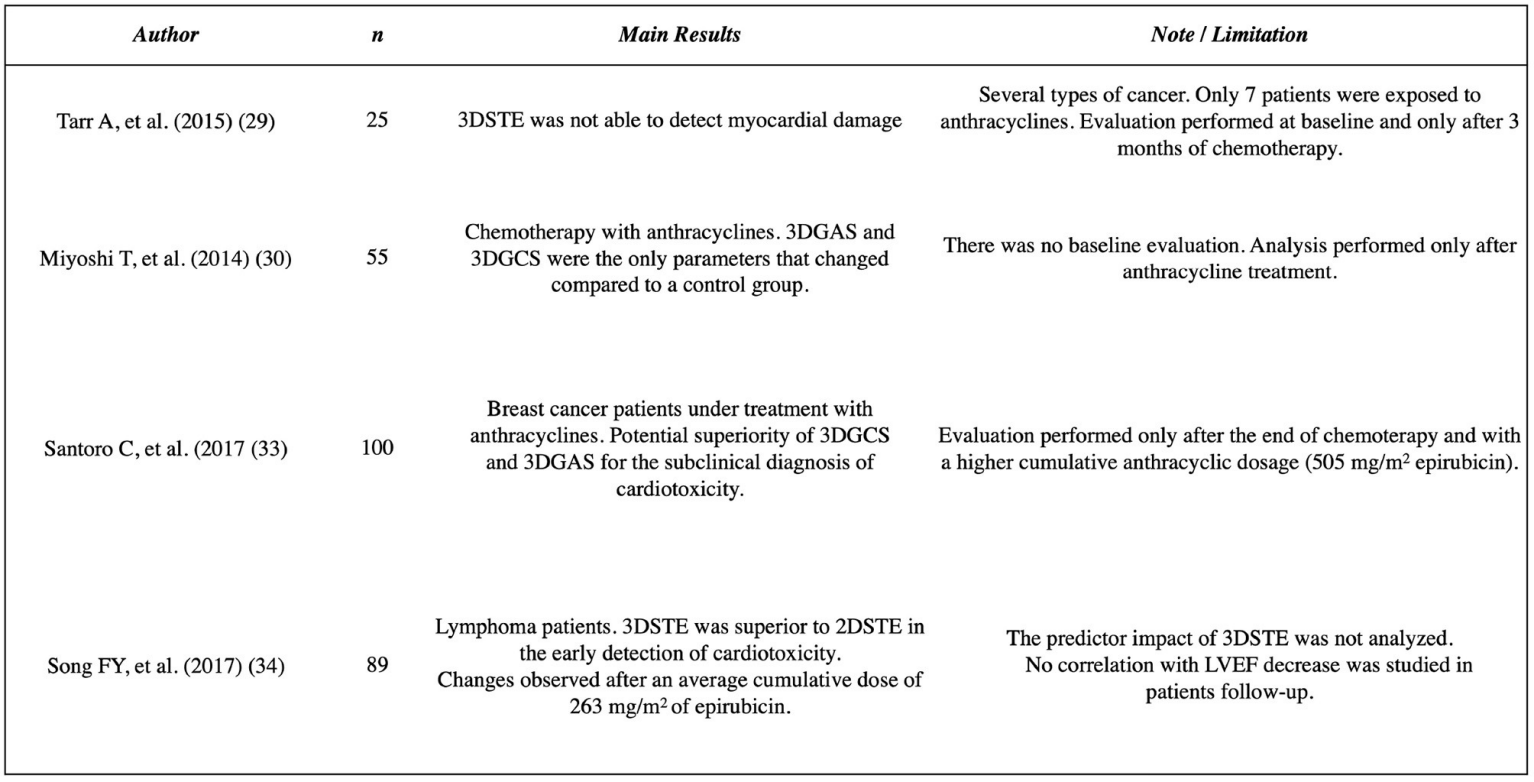
Studies reporting the 3D speckle tracking echocardiography analysis in cancer patients undergoing chemotherapy. GAS, global area strain; GCS, global circumferential strain; LVEF, Left ventricle ejection fraction. STE, speckle tracking echocardiography.

In our evaluation 3DGAS was the only parameter that changed at an earlier stage, after a lower cumulative dosage of doxorubicin (120 mg/m^2^). In addition, most importantly, the 3DGAS changed to below normal values was the only parameter that was associated with the subsequent decrease in LVEF during follow-up (Figure 5). These data suggest the superiority of this parameter for early detection of CTRCD. Area strain is a new 3DSTE index that has demonstrated applicability in different clinical scenarios; it allows a relatively operator-independent quantitative evaluation of LV function and combines longitudinal and circumferential deformations analysis, probably being more sensitive in detecting anomalies specially in the subendocardium layer, one of the earliest affected areas in several heart conditions (32). In the setting of cardiotoxicity, recent research has evaluated 100 patients with breast cancer under treatment with anthracyclines and demonstrated a potential superiority of 3DGCS and 3DGAS in comparison to other echocardiographic parameters for the subclinical diagnosis of cardiotoxicity. However, the evaluation of these patients was performed after a higher cumulative anthracyclic dosage (mean of 505 mg/m^2^ of epirubicin) (33). Another recent study evaluated 89 patients with lymphoma; in comparison to 2DSTE, 3DSTE was superior in the early detection of cardiotoxicity. In this research, the changes were observed after an average cumulative dose of 263 mg/m^2^ of epirubicin (34). In Figure 6, we have highlighted the main findings of these studies that involve 3DSTE and cancer patients who underwent chemotherapy.

Another point to be highlighted in our study was the evidence of good reproducibility of 3DSTE, especially in 3DGAS and 3DGCS, confirming that they are reliable and accurate indices in the assessment of ventricular mechanics.

The 3DGAS represents a new automatic promissory index, with a more accurate analysis of global and regional left ventricular systolic function. The 3DGAS mainly includes the assessment of myocardial deformation in the subendocardial and endocardial layers (18). The occurrence of cellular oxidative stress, the main pathophysiological mechanism of cardiotoxicity, leads to different degrees of myocyte dysfunction (35). Studies in animal models using 2DSTE and cellular oxidative stress markers have shown that myocyte apoptosis, capillary density reduction, inflammatory response in cardiac tissue, and myofilament degradation promote early changes in myocardial deformation, before the decrease in LVEF and LV fractional shortening (36). In our study, the early change in 3DGAS suggests that early oxidative stress injury can affect the different myocardial layers at a very early stage.

Myocardial deformation indices assessed by 2DSTE have played an important role in the early detection of subclinical left ventricular dysfunction in patients who underwent chemotherapy (8–11, 15). The main parameter involved is the 2DGLS, and a relative percentage reduction >12–15% from baseline is very likely to be abnormal (10, 12, 13, 27). In our study, a significant reduction of 2DGLS after a cumulative dosage of 240 mg/m^2^ of doxorubicin was observed. However, the relative change of 2DGLS was mild (only 0.7%), with no patient presenting criteria for the diagnosis of subclinical cardiotoxicity (25, 26). Notably, after a low dosage of doxorubicin (120 mg/m^2^), there was no significant reduction in 2DGLS. There are little data on the evaluation of these patients after exposure to lower doses of anthracyclines. In a recent study that involves 86 patients, 2DGLS was evaluated after cumulative doxorubicin dosages of 150 mg/m^2^ (slightly higher than our study). In this series, 2DGLS was a predictor of cardiotoxicity in the 6 (7%) patients who were presented with LVEF reduction after 1 year of cancer treatment, showing the importance of evaluating the patient at an earlier time (37). In this study, a few patients were exposed also to radiotherapy, increasing the risk of cardiotoxicity; in addition, as the number of patients who developed cardiotoxicity was too small, these data should be confirmed in a larger population sample. Regarding 2DGRS, this parameter did not change after treatment with anthracyclines, corroborating previous studies that demonstrated its low sensitivity for detection of chemotherapy-induced cardiotoxicity (8, 10). Two-dimensional GCS was not evaluated in the present study, considering previous reports that revealed its low reproducibility and less relevance in the scenario of cardiotoxicity (12).

Despite the promising and important results related to the early diagnosis of cardiotoxicity through the 2DSTE, the benefit of cardioprotective treatment based exclusively on the decrease in the 2DGLS is not yet well established. The 1-year results of Strain Surveillance of Chemotherapy for Improving Cardiovascular Outcomes (SUCCOUR) trial were recently reported (27). This was the first prospective randomized, open, blinded end-point assessment study designed to compare the cardioprotective treatment (beta-blockers plus either angiotensin-converting enzyme inhibitors or angiotensin receptor blockers) guided by a decrease in LVEF (>10% to an absolute value <55% or if LVEF drop by 5% accompanied by symptoms) vs. decrease in 2DGLS (relative drop ≥12%). The authors observed that 2DGLS-guided cardioprotective treatment significantly reduced a meaningful fall of LVEF to the abnormal range, supporting the use of 2DSTE in surveillance for CTRCD. However, the study was failed to meet its primary endpoint—the change in LVEF was not different between the two arms. Furthermore, no clinical outcomes were observed (definitive impact still unclear). Additional results are therefore necessary to establish this approach, especially when we consider that unnecessary cardiovascular treatment and inappropriate interruption of cancer treatment can be observed in this context (38). In our study, during chemotherapy with anthracyclines, patients did not have a drop in 2DGLS with criteria of subclinical cardiotoxicity that would justify cardioprotective treatment. We did not observe HF or unfavorable cardiovascular clinical outcomes (probably due to the short follow-up time), limiting the assessment of the clinical impact related to the early decrease in 3DGAS. Patients who had a decrease in LVEF with cardiotoxicity criteria during follow-up were treated with beta-blockers plus either angiotensin-converting enzyme inhibitors or angiotensin receptor blockers.

No changes were observed in the conventional Doppler indices. In fact, the use of these parameters as early markers of cardiotoxicity is questionable, since they are considerably influenced by load conditions (12).

Troponin I can predict the development of later cardiac events in patients who were treated with high doses of anthracyclines or chemotherapy with trastuzumab, and it is a sensitive and specific marker for myocardial injury in adults who underwent chemotherapy (39). In the present study, after the cumulative dosage of 240 mg/m^2^ of doxorubicin, 45% of the patients presented abnormal levels of this biomarker. However, there was no early change in US-TnI, after a lower cumulative dose of doxorubicin (120 mg/m^2^). Most importantly, US-TnI was not associated with a subsequent decrease in LVEF (*p* = 0.684). Currently, some challenges remain regarding the widespread application of troponin, such as determining the optimal timing of troponin assessment and defining the cutoff point to define cardiotoxicity.

Early identification of chemotherapy-induced cardiotoxicity is critical to reduce morbidity and mortality in the oncologic population. In this context, adequate clinical risk stratification (identification and control of cardiovascular risk factors and knowledge of the cardiotoxic potential of cancer treatment) is essential (40). Cardiovascular imaging methods will help the pre-treatment risk stratification, in addition to being fundamental in monitoring during and after chemotherapy (41). The 3DSTE, in particular 3DGAS, has the potential to be a future tool incorporated into risk stratification algorithms and can add value in the early recognition of a population at higher risk for the subsequent development of CTRCD.

The clinical implications of the present study are important. The reported findings demonstrate that 3D strain, a non-invasive and easily performed analysis, recognizes subtle myocardial damage and predicts a later decrease in the LVEF in patients receiving anthracyclines.

## Limitations

The study comprised a small sample size and the echocardiographic and laboratory evaluations were performed in a single research center; multicenter studies and a larger population sample are required to validate tour results. Moreover, a longer-term follow-up study would be necessary to determine whether early changes in 3DGAS could correlate with clinical outcomes. In addition, the drop in LVEF was slight and no patient had symptoms of HF. Thus, targeted studies evaluating the comparison of cardioprotective treatment guided by a change in 3DGAS against this treatment guided by a decrease in LVEF are needed to determine the real prognostic impact of this tool. For 2DSTE, we performed the radial strain analysis only from the short-axis mid-papillary level, thus, not representing global radial strain. There was a significant sample loss during patient follow-up.

## Conclusion

In patients with breast cancer who were treated with anthracyclines, an analysis of myocardial mechanics using 3DSTE detected early changes in 3DGAS after very low doses of doxorubicin. The 3DGAS early changed to abnormal values was associated with a subsequent decrease in LVEF, representing a promising technique to predict chemotherapy-induced cardiomyopathy.

## Data Availability Statement

The original contributions presented in the study are included in the article/supplementary material, further inquiries can be directed to the corresponding author/s.

## Ethics Statement

The studies involving human participants were reviewed and approved by Comitê de ética em pesquisa do Hospital Israelita Albert Einstein. The patients/participants provided their written informed consent to participate in this study.

## Author Contributions

AR and MV: conception and design, acquisition, analysis and interpretation of data, revising the article critically for important intellectual content, and final approval of submitted version. CF: conception and design, acquisition of data, drafting the article, and final approval of submitted version. TA: acquisition of data, drafting the article, and final approval of submitted version. ED, FC, TG, and MK: conception and design, draft the article, and final approval of submitted version. SM: conception and design, interpretation of data, drafting the article, revising the article critically for important intellectual content, and final approval of the submitted version. All authors contributed to the article and approved the submitted version.

## Conflict of Interest

The authors declare that the research was conducted in the absence of any commercial or financial relationships that could be construed as a potential conflict of interest.

## Publisher's Note

All claims expressed in this article are solely those of the authors and do not necessarily represent those of their affiliated organizations, or those of the publisher, the editors and the reviewers. Any product that may be evaluated in this article, or claim that may be made by its manufacturer, is not guaranteed or endorsed by the publisher.

## Abbreviations

2DGLS: Two-dimensional global longitudinal strain
2DRS: Two-dimensional radial strain at midpapillary level
2DSTE: Two-dimensional speckle tracking echocardiography
3DGAS: Three-dimensional global area strain
3DGCS: Three-dimensional global circumferential strain
3DGLS: Three-dimensional global longitudinal strain
3DGRS: Three-dimensional global radial strain
3DSTE: Three-dimensional speckle tracking echocardiography
ACE: Angiotensin-converting enzyme
ARB: Angiotensin II receptor blocker
CMR: Cardiac magnetic resonance
CTRCD: Cancer-therapeutic related cardiac dysfunction
HF: Heart failure
LVEF: Left ventricular ejection fraction
TAPSE: Tricuspid annular plane systolic excursion
US, TnI: Ultrasensitive troponin I.
